# Complex Greenland outlet glacier flow captured

**DOI:** 10.1038/ncomms10524

**Published:** 2016-02-01

**Authors:** Andy Aschwanden, Mark A. Fahnestock, Martin Truffer

**Affiliations:** 1 University of Alaska Fairbanks, Fairbanks, Alaska, USA

## Abstract

The Greenland Ice Sheet is losing mass at an accelerating rate due to increased surface melt and flow acceleration in outlet glaciers. Quantifying future dynamic contributions to sea level requires accurate portrayal of outlet glaciers in ice sheet simulations, but to date poor knowledge of subglacial topography and limited model resolution have prevented reproduction of complex spatial patterns of outlet flow. Here we combine a high-resolution ice-sheet model coupled to uniformly applied models of subglacial hydrology and basal sliding, and a new subglacial topography data set to simulate the flow of the Greenland Ice Sheet. Flow patterns of many outlet glaciers are well captured, illustrating fundamental commonalities in outlet glacier flow and highlighting the importance of efforts to map subglacial topography. Success in reproducing present day flow patterns shows the potential for prognostic modelling of ice sheets without the need for spatially varying parameters with uncertain time evolution.

## Abstract (web summary)

Quantifying Greenland's future contribution to sea level requires accurate portrayal of its outlet glaciers in ice sheet simulations. Here, the authors show that outlet glacier flow can be captured if ice thickness is well constrained and vertical shearing as well as membrane stresses are included in the model.

## Graphical abstract

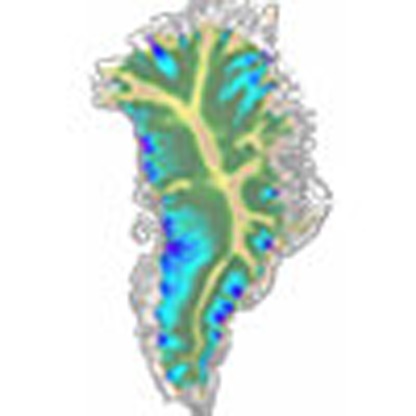

High spatial variability in the flow of the Greenland Ice Sheet is apparent from observations^1^, and capturing this variability is essential to any modelling effort targeting the future evolution of the Greenland Ice Sheet^2^, yet projections of ice discharge into the ocean remains a major wild card for twenty first century sea-level projections^3^,^4^. Ice flow is nonlinearly related to ice thickness, with small uncertainties in ice thickness potentially leading to large biases in discharge estimates^5^. Consequently, our ability to reproduce observed flow patterns, and thus ice discharge, is contingent on accurate knowledge of ice thickness; however, limited knowledge of thickness has hindered modelling efforts to date. In Greenland, the NASA airborne mission Operation IceBridge (OIB)^6^ has added many 1,000 km of radar-derived ice thickness profiles since 2009, nearly doubling the coverage available at that time. To help fill in remaining gaps, mass-conserving interpolation methods^7^ have been used to derive flow-compatible, high-resolution maps of ice thickness and subglacial topography^8^. The result has been a substantial improvement in our knowledge of subglacial topography, particularly in the deep channel-feeding outlet glaciers. At the same time, code parallelization, combined with high-performance computing, have begun to make high-resolution ice sheet modelling tractable.

Combining these advances allows us to pursue a set of numerical experiments to investigate whether spatially complex flow patterns in outlet glaciers can now be captured in whole-ice-sheet simulations using only ice-sheet-wide (spatially uniform) parameters, without local ‘tuning' applied to individual grid cells.

Here we use the Parallel Ice Sheet Model (PISM)^9^, a computationally efficient, three-dimensional model^10^,^11^, coupled with models of subglacial hydrology and basal sliding, to simulate the velocity field of the Greenland Ice Sheet at high resolution (<1 km). We demonstrate that outlet glacier flow can be captured with high fidelity if ice thickness is well constrained and vertical shearing as well as membrane stresses are included in the model (without solving the full-stress configuration), while computing flow from vertical shearing alone and/or using low-resolution ice thickness leads to poor agreement with observations. Overall root mean squared (r.m.s.) velocity differences decrease with increasing model resolution. This indicates that ongoing improvements in the mapping of subglacial topography, together with improvements in modelling resolution, go a long ways towards improved whole-ice-sheet numerical simulations.

## Results

### Calibration and validation

Using the ice sheet geometry given by ice thickness and subglacial topography from Morlighem *et al.*^8^ (data set MO2014), we calculated diagnostic velocity fields with PISM. We calibrated the model at a horizontal grid resolution of 1,500 m by using 15 runs to explore the parameter space defined by three spatially uniform parameters controlling ice dynamics and basal processes, selecting the run with the best fit to observed outlet glacier flow (see Methods and Supplementary Table 1).

**Figure 1:**
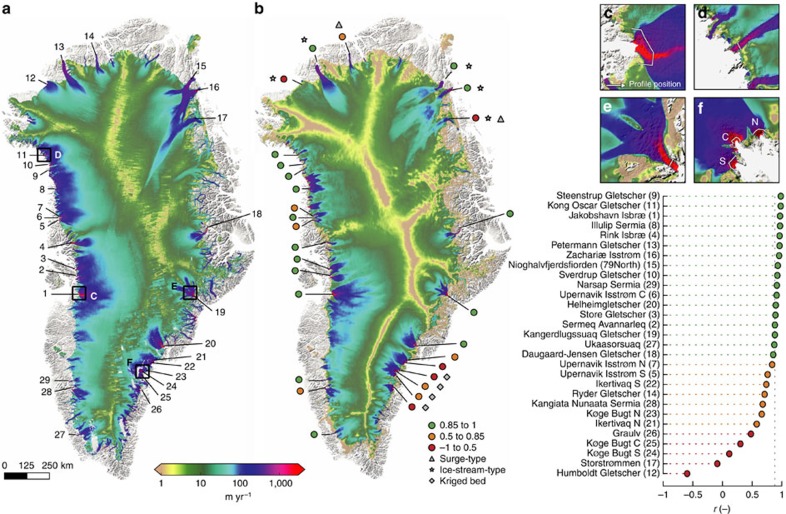
Surface speeds for Greenland for 2008–2009. (**a**) Observed speeds^12^, adjusted to represent annual averages. (**b**) Calibrated model speeds at 600-m grid resolution. Coloured dots indicate Pearson's *r* correlation coefficient and the dotted grey vertical line indicates the median value (0.88). (**c**–**f**) Inset showing simulated surface speeds of Jakobshavn Isbræ (**c**), Kong Oscar Gletscher (**d**), Kangerdlugssuaq Gletscher (**e**) and Køge Bugt (**f**). White lines indacte the position of the profile. Speeds are masked where observed ice thicknesses are <50 m.

We assessed model performance by comparing observed and simulated surface velocities along cross-flow profiles of 29 large (flux ≥∼3 Gt yr^−1^) outlet glaciers (Fig. 1a, Supplementary Table 2), which account for ∼2/3 of the ice-sheet discharge (∼520 Gt per year in 2008)^4^. Along these profiles, we sampled remotely sensed^12^ and modelled surface velocities every 250 m using bilinear interpolation. We quantified the model's ability to capture the spatial variation in flow structure using the Pearson *r* correlation coefficient and the r.m.s. difference between observations and simulations along the 29 profiles. The correlation coefficient provides an indication of the model's ability to capture the velocity variation along a profile (related to the channelization of flow), while the r.m.s. value captures the ability of the model to match the magnitude of the velocity, indicating how well the model reproduces discharge flux of each glacier.

Within the tested parameter space, 9 of 14 calibration experiments are within 25% of the smallest total r.m.s. difference (that is, the r.m.s. difference between observed and simulated velocity profiles summed over all outlet glaciers, see Supplementary Table 3); and all but one median correlation coefficient is larger than 0.74 (Supplementary Table 3). Observed horizontal surface velocities increase from near-zero at ice divides to ∼10 km per year in outlet glaciers, covering many orders of magnitude. This overall pattern is well represented in all calibration runs (Supplementary Fig. 1) where flow arises due to the sum of longitudinal stretching and vertical shearing. For comparison, in a simulation where ice flow is only due to vertical shearing (see Methods), outlet glacier flow is basically absent (Supplementary Fig. 2). This suggests that the spatial variability in flow can be explained to a large degree by the variability in ice thickness if membrane stresses are included.

With the calibrated model we performed simulations over a range of horizontal grid resolutions from 4,500 to 600 m. This allows us to address the impact of model resolution on simulation of flow in outlet glaciers, helping to set limits on the minimum resolution required to capture such flow.

### Reproduction of flow pattern at the highest model resolution

Of the 29 analysed outlet glaciers, the flow of 4 is characterized by low-surface slopes and low driving stresses (‘ice-stream'-type), while all other glaciers flow through channels significantly deeper than the surrounding ice (‘isbræ'-type)^13^. At our highest model resolution (600 m) the calibrated model reproduces the velocity structure of ‘ice-stream' type glaciers slightly better on our cross profiles (median *r*=0.93) than ‘isbræ' type glaciers (median *r*=0.88). Flow speeds in the transitional zone 100–300 km inland (speeds approximately 20–100 m per year) are generally underestimated (Fig. 1a,b).

Along the cross-flow profiles, simulated flow structure agrees well (*r*>0.85) with observations for 17 of the 29 glaciers, agrees fairly well for 7 (0.5≤*r*≤0.85) and agrees poorly (*r*<0.5) for only 5, with a median of 0.88 (Fig. 1b–f).

Of the 14 fast-flowing marine-terminating glaciers with a well-represented flow structure (*r*>0.85), the simulated surface speeds of 4 are close to observed speeds (Daugaard–Jensen Gletscher, Narsap Sermia, Sermeq Avannarleq and Ukaasorsuaq), 8 are too low (Helheimgletscher, Jakobshavn Isbræ, Kangerdlugssuaq Gletscher, Kong Oscar Gletscher, Rink Isbræ, Steenstrup Gletscher, Store Gletscher, Sverdrup Gletscher) and the speeds of only 2 (Illulip Sermia and Upernavik Isstrøm C) are too high.

Two glaciers, Ryder Gletscher (*r*=0.71) and Storstrømmen (*r*<0), are surge-type glaciers whose flow shows large variations over time due to hydrologically controlled sliding^14^,^15^, largely uncoupled from surface slope and ice thickness, which may have led to a reduced fit. In addition, Graulv and the Køge Bugt glaciers have correlation coefficients less than or equal to 0.65, substantially lower than the median (0.88). For these glaciers, Morlighem *et al.*^8^ used kriging to derive ice thicknesses away from OIB measurements, while they used mass conserving interpolation techniques^7^ for all other glaciers, enabled by better OIB measurement coverage. We hypothesize that the poor match between simulated and observed flow for these glaciers results from poorly constrained ice thickness knowledge. While glaciers are rapidly changing, our model is diagnostic and should reflect the flow for the current geometry. Results for other glaciers with large dynamic adjustments, such as Jakobshavn Isbræ, are very encouraging.

### Role of model resolution

**Figure 2:**
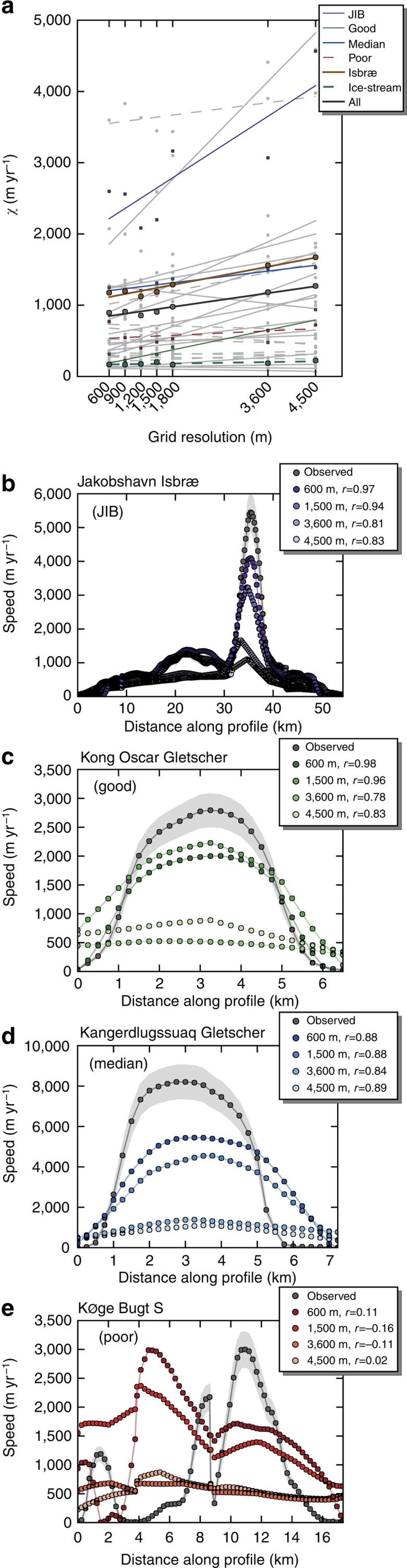
Outlet glacier profiles as a function of grid resolution. (**a**) Linear regression analysis of the root mean square (r.m.s.) difference *χ* under grid refinement. Dots mark total *χ*, and solid and dashed lines indicate statistically significant and not significant trends, respectively. Also, the trends of all glaciers, all ‘isbræ'-type, and all ‘ice-stream' type glaciers are shown. (**b**–**e**) Four examples of observed and simulated surface velocity cross profiles, evaluated every 250 m along profiles using bilinear interpolation; Jakobshavn Isbrae (JIB) (**b**), a glacier that has a high correlation coefficient (Kong Oscar Gletscher) (**c**), the glacier whose correlation coefficient represents the sample median (Kangerdlugssuaq Gletscher) (**d**) and a glacier with a poor fit (Køge Bugt S) (**e**). The shaded grey area indicates observational uncertainty.

**Figure 3:**
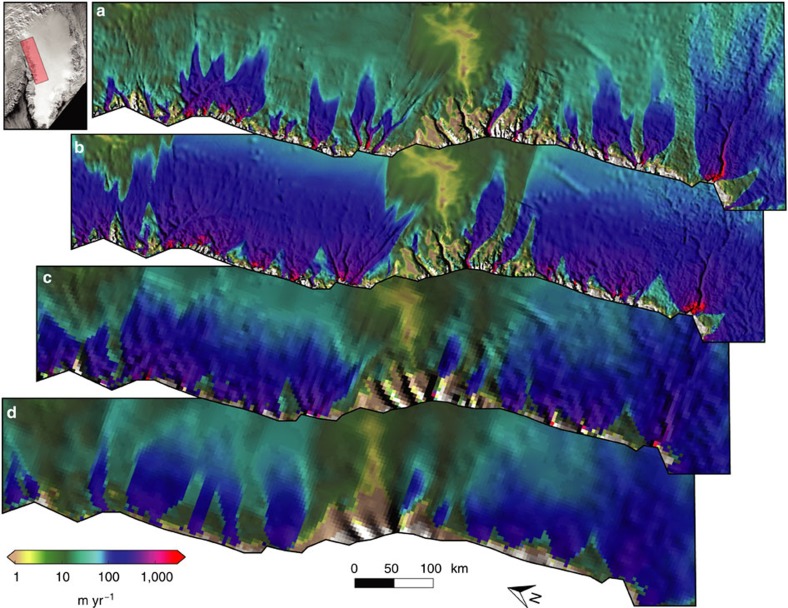
Simulated surface speeds for the northwest coast of Greenland. (**a**) Overview. (**b**) 600-m grid resolution and MO2014 subglacial topography. (**c**) 4,500-m grid resolution and MO2014 subglacial topography. (**d**) 600-m grid resolution and BA2001 bed topography. (**e**) 4,500-m grid resolution and BA2001 subglacial topography. Background: shaded relief of the respective subglacial topography.

The impact of poor model resolution on the simulation of outlet glacier flow is illustrated by resampling the MO2014 subglacial topography and ice thickness to a range of model grid spacings (Fig. 2). To quantify the impact of improved model resolution, we performed a linear regression analysis of the r.m.s. velocity difference for each glacier. A simulation at the lowest model resolution (4,500 m) has an r.m.s. velocity difference 42% higher than at 600 m (Fig. 3c). Overall we find that r.m.s. velocity differences decrease with increasing resolution (goodness-of-fit *r*^2^=0.95). Sixteen glaciers have statistically significant (*P*<0.05) improving trends. All but one of these (Humboldt Gletscher) are fast-flow marine-terminating glaciers (mean velocity >200 m yr^−1^) of the ‘isbræ'-type. Jakobshavn Isbræ, Greenland's most prolific supplier of ice to the ocean, flows through a <10-km wide subglacial valley grounded well below sea level. A minimum resolution of ∼2 km is needed to resolve the valley geometry sufficiently to reproduce the high (>2 km yr^−1^) surface velocities (Fig. 2b, Supplementary Fig. 3). ‘Ice-stream'-type glaciers and surge-type glaciers (Ryder Gletscher and Storstrømmen) and ‘isbræ'-type glaciers with subglacial topographies derived by kriging (Graulv, Køge Bugt N and S; Supplementary Figs 3–5) do not benefit from increased resolution.

### Role of ice thickness

To assess how much OIB measurements and mass-conserving interpolation methods have improved model simulations, we performed two simulations with an older ice thickness/subglacial topography data set (BA2001)^16^. The BA2001 data set is posted at 5,000 m, which we resampled to 600 and 4,500 m spacing. For these experiments, the total r.m.s. difference between modelled and observed flow of all 29 outlet glaciers are 49% (600 m) and 57% (4,500 m) higher than with the MO2014 ice thickness data set at 600-m resolution (Fig. 3, Supplementary Table 4). At the same time, the reproduction of the flow structure is substantially better when using the MO2014 data set at a model resolution of 600 m (median correlation coefficient 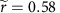) than using the BA2001 data set (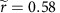 and 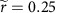 at 600 and 4,500 m model resolution, respectively). High model resolution alone clearly is insufficient to reproduce outlet glacier flow (Supplementary Figs 6–8) Furthermore resampling data sets to resolutions higher than the nominal resolution (for example, resampling the BA2001 data set to 600 m) does not generate physical information that is not already captured by the coarse data. We note that, in some cases, such resampling may produce an apparent improvement. However, this improvement is an artifact of the analysis method (Supplementary Note 1).

### Role of flow type

Of Greenland's 200+ outlet glaciers^1^, only 4 are ‘ice-stream'-type whereas the rest are ‘isbræ'-type. While our selection of 29 outlet glaciers includes all four ‘ice-stream'-type glaciers, any statistical analysis with only four samples requires caution.

The reproduction of the flow structure of ‘isbræ'-type glaciers at 600 m model resolution is substantially better (median correlation coefficient 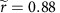) for the MO2014 data set than for BA2001 (0.54) at the same model resolution, as well as at 4,500 m model resolution (0.09). Also the r.m.s. velocity difference is much lower.

Compared to ‘isbræ'-type glaciers, ‘ice-stream'-type glaciers are less sensitive to both model and data set resolution. Furthermore, the total r.m.s. velocity differences of ‘ice-stream'-type glaciers is almost an order of magnitude smaller than for ‘isbræ'-type glaciers (Supplementary Table 4).

The structures of three ‘ice-stream' type glaciers that are well represented at the highest resolution (79North, Petermann Gletscher and Zachariæ Isstrøm) are well represented at all grid resolutions. The improving trend of ‘ice-stream'-type glaciers is not statistically significant (Fig. 2a). This suggests that all grid resolutions sufficiently capture lateral variations in ice thickness and surface slope, which are typically small in ‘ice-stream' type glaciers. For ‘ice-stream'-type glaciers accurate knowledge of ice thickness is less crucial to reproduce the flow structure than it is for ‘isbræ'-type glaciers. Nonetheless subglacial topography still needs to be well constrained to properly treat advance and retreat scenarios.

### Humboldt Gletscher

Humboldt Gletscher stands out in all aspects. Located in the northwestern corner of Greenland, the ∼90-km wide ‘ice-stream' type glacier has a mean velocity <200 m yr^−1^. Humboldt's simulated flow structure agrees poorly with observations at all resolutions, shows the smallest improving trend, and has the lowest goodness-of-fit (*r*^2^=0.63). Simulated basal temperatures for Humboldt are very close to the pressure-adjusted melting point, and depending on grid resolution, basal motion may be activated or not in our simulations. The flow of Humboldt Gletscher is thus very sensitive to grid resolution and model parameters, unlike other glaciers studied. To explain this sensitivity, a more focused study might be warranted, possibly using time-dependent data assimilation and inverse methods such as in refs 17, 18.

## Discussion

The simulations presented in this study portray Greenland's flow structure in unprecedented detail; most large outlet glaciers considered are well-captured at grid resolutions <1 km. Our results demonstrate that spatial variability in flow can be explained in large part by the spatial variability in ice thickness. We find that inversion of surface properties for individual glaciers is not essential to reproduce the overall flow pattern. Using simple parametrizations of basal motion and subglacial hydrology, we find good agreement between simulated and observed spatial flow patterns while the reproduction of the velocity magnitude requires further improvements, especially in the transitional zone 100–300 km inland (speeds ∼20–100 mper year). Disagreement between observed and simulated speeds may arise from inadequacies in parametrizing basal motion and subglacial hydrology. Also not all heat sources that affect the viscosity of ice are accounted for in our model. For example refreezing of surface meltwater (‘cryo-hydrologic warming') can soften the ice and enhance flow, which may be relevant in the transitional zone^19^,^20^,^21^,^22^. Furthermore the ice flux from the interior may be misrepresented because interior ice thickness remains less well characterized, but might be just as important to capture outlet glacier flow downstream. Finally, we calibrated only three parameters and only at the locations of our profiles, which may partly explain the reduced fit in the interior. To further reduce the gap between observations and simulations, future work must elucidate the physics behind the remaining misfit between modelled and measured flow across the spectrum of outlet systems, in the transitional zone and the interior. A quantitative, physically based understanding of the mismatch can then guide future modelling efforts.

Here we have presented diagnostic simulations; future work should address whether the observed temporal variability can be explained by our simple parametrizations or whether more physically based models of subglacial hydrology are needed. Basal motion can be highly variable locally on short (<1 year) time scales^23^,^24^,^25^,^26^, arising from variations in the hydrological system. Development of adequate models of subglacial hydrology, applicable on an ice sheet wide scale, is still at an early stage (for example, ref. 27). On a regional scale, recent efforts show promise in capturing the seasonal evolution in flow speeds resulting from changes in basal resistance caused by surface meltwater delivery^28^.

Here we have used surface velocities as our metric of success. It could be argued that ice discharge is a more suitable metric of success. However, ice thickness is subject to larger observational uncertainties than ice velocity, making discharge (the product of ice thickness and vertically averaged horizontal velocities) a weaker metric. We note that our results would look even more favourable if we used flux as a metric, with 20 glaciers showing correlation coefficients exceeding 0.85 (Supplementary Fig. 9), and the fluxes of 6 marine-terminating glaciers being within observational uncertainty.

Clearly, large-scale observational efforts such as OIB improve our ability to model the parts of the system that are responsible for the bulk of the mass flux to the oceans. The simple metrics we introduced here help to quantify the impact of these additional measurements on whole ice-sheet simulations. Ice thickness measurements by many groups are ongoing and OIB, currently scheduled to continue until 2019, is expected to add many line kilometres of measurements by that time. Improvements such as those discussed here for Greenland would also be expected from additional investment in mapping subglacial topography for the Antarctic ice sheet.

Our simulations have implications for efforts targeted at projecting twenty first century sea level rise. IPCC's Fourth Assessment Report called out the need to resolve the full stress configuration in ice-sheet models to simulate changes in outlet glaciers. Since then, ice sheet models have seen substantial improvements in their representation of flow physics (for example, refs 29, 30). We find that models that resolve both membrane and vertical stress gradients are capable of reproducing the observed flow structure with high fidelity. In regions with large transverse velocity gradients, such as shear margins, the mismatch between observed and simulated flow may be further reduced by resolving additional components of the stress balance.

The large improvements we have attained using only spatially uniform parameters suggest that there is much to be learned about outlet glacier flow from comparing suites of glaciers to each other. By modelling each outlet glacier with the same spatially uniform parameters, modelled flow variations are dependent locally only on the shape of the bed and surface. Discordance with observed velocity, if not due to errors in these shapes, then may reflect departure from the conditions assumed by these uniform parameters. If groups of glaciers that experience similar surface melt and accumulation patterns show similar departures, this would indicate a common response that is not captured by present physics. In this way, it should be possible to limit the degrees of freedom in the model as the parameter space is enhanced to better capture spatial patterns of flow. This type of analysis would not be well bounded or even tractable if locally variable parameters had been used to match the observed flow patterns of each glacier.

Successful prognostic models will have to demonstrate that both spatial and temporal variability in flow can be replicated^2^; here, we have shown significant progress in the former.

## Methods

### Outlet glaciers

Along cross-profiles of 29 outlet glaciers (Supplementary Table 2) we sample observed and modelled surface velocities every 250 m using bilinear interpolation, and all metrics are then calculated from those profiles. Profiles are drawn roughly perpendicular to observed flow directions, following flightlines whenever possible. Observed surface velocities^12^ **U**_s_ represent fall velocities of 2008; we assume that annually-averaged velocities **U** are 10±5% higher than fall speeds, **U=**1.1**U**_s_ (ref. 26).

The flow component normal to the gate is given by *U*_⊥_=**n**·**U**, where **n** is the unit normal vector at each gate point such that **n**·**U** is positive. We calculate the uncertainty in surface velocities, σ, by adding 0.05*U*_⊥_ to the 1-sigma error of the data set, *U*_e_:


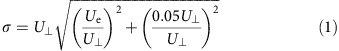


### Ice sheet model

Simulations are performed with the open-source Parallel Ice Sheet Model (PISM), which is thermomechanically coupled, polythermal, and includes a hybrid stress balance model^10^,^31^. The hybrid scheme combines the Shallow Ice Approximation (SIA)^32^ for vertical deformation and the Shallow Shelf Approximation (SSA)^33^ for longitudinal stretching. The effective viscosity of glacier ice, *η*, is given by


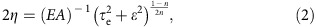


where *E* is the flow enhancement factor, *τ*_e_ is the effective stress, *A* is the enthalpy-dependent rate factor (softness), and *n* is the exponent of the power law. The small constant *ɛ* (units of stress) regularizes the flow law at low effective stress, avoiding the problem of infinite viscosity at zero deviatoric stress.

### Boundary conditions

At the basal boundary, geothermal flux varies in space^34^, and a pseudoplastic power law^35^ relates bed-parallel shear stress, **τ**_b_, and the sliding velocity, **u**_b_:


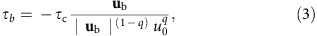


where *τ*_*c*_ is the yield stress, *q* is the pseudoplasticity exponent, and *u*_0_=100 m yr^−1^ is a threshold speed. Here we use a simple model of basal hydrology that connects basal water pressure and the rheology of soft sediments (‘undrained plastic bed')^36^. While this model was motivated by observations from Ice Stream B in Antartica^37^, there is growing evidence of soft sediments beneath the Greenland Ice Sheet^38^,^39^,^40^,^41^. Furthermore, theoretical work^42^ suggests that hard beds, that is beds without soft sediments present, behave plastically at high basal water pressure. We assume that yield stress *τ*_c_ is proportional to effective pressure *N* (‘Mohr-Coulomb criterion'^43^),


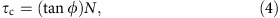


where *φ* is the till friction angle and the effective pressure *N* is given by^37^,^27^:


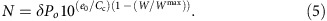


Here *δ*=0.02 is a lower limit of the effective pressure, expressed as a fraction of overburden pressure, *e*_0_ is the void ratio at a reference effective pressure *N*_0_, *C*_c_ is the coefficient of compressibility of the sediment, *W* is the effective thickness of water and *W*^max^ is the maximum amount of basal water. We use a non-conserving hydrology model that connects *W* to the basal melt rate 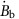 (ref. 37):


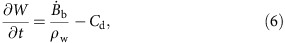


where *ρ*_w_ is the density of water and *C*_d_=1 mm yr^−1^ is a fixed drainage rate.

We prescribe the yield stress as a function of bed elevation^44^ by prescribing the till friction angle *φ* as a continuous function of the bed elevation, with *φ*=5° for bed elevations lower than 700 m below sea-level, *φ*=40° for bed locations higher than 700 m above sea-level, and changing linearly in between. While the bed elevation dependence is not derived strictly from first principles, outlet glaciers with bed elevations below sea-level have hydrologic systems tied to sea level at the terminus, and so run at high basal water pressure (that is, low effective pressure), and may be underlain by marine sediments^45^, which are weak^46^.

To assess the role of this bed elevation dependence, we performed a simulation where the yield stress is only a function of locally derived effective pressure, not dependent on bed elevation. In this simulation, fast flow is apparent in outlet glaciers; however, not of the right structure (Supplementary Figs 10–12). The total (all 29 outlet glaciers) r.m.s. difference is 27% higher than the calibrated simulation, indicating that using an elevation-dependent yield stress provides a much improved match to the flow structure of most investigated glaciers.

### Model initialization

The initialization procedure follows closely the ‘flux-corrected paleo-climate' initialization method in ref. 47. During the last 5,000 years of the initialization we apply a flux-correction method to obtain an ice sheet geometry in closer agreement with measurements^47^. For computational efficiency grid refinements are made during the initialization procedure. The runs are started on a 18,000 m grid at −125,000 years, then refined from 18,000 to 9,000 m at −25,000 years, to 4,500 m at −5,000 years, to 3,600 m at −1,000 years, to 1,800 m at −500 years, to 1,500 m at −300 years, to 1,200 m at −200 years, and finally to 900 m at −100 years. The vertical model resolution is 20 m.

### Model calibration

We calibrate three ice-sheet-wide scalar parameters, the flow enhancement factor *E*, the exponent of the sliding law *q*, and the exponent of the flow law for the SSA *n*, while all other parameters are PISM default. The calibration is performed at 1,500-m grid resolution.

To calculate the velocity field that corresponds to a given parameter combination, one could solve the non-linear system of equations of steady state using the observed geometry and standard direct or iterative solution techniques. Without smoothing applied to the observed geometry, such a velocity field would reflect any inconsistencies between the calculated thermodynamic and hydrological initial state and observations. Instead we attempt to follow the physical transient using a flux correction method^47^ to iterate towards a geometry that is ‘close' to the observed geometry and is consistent with the model and the applied forcing. Such a flux correction is used to minimize the difference between the modelled and the observed geometry. All experiments use the enthalpy field from the end of the initialization procedure at the corresponding grid resolution, except for 600-m grid resolution, which starts from the initialized state at 900-m grid resolution. All experiments are run for 100 years, sufficiently long to adjust to a given parameter combination. Flux correction does not respect observed ice thickness perfectly; especially in outlet glaciers near the grounding line, differences may locally exceed 100 m.

First we calibrate *E* by running the model in SIA-only mode and comparing simulated with observed surface speeds in slow-flowing (<20 m yr^−1^) areas. The r.m.s. differences between modelled and observed surface speed range from 6 m yr^−1^ (*E*=1.5) to 12 m yr^−1^ (*E*=3; Supplementary Fig. 13). Except for *E*=3, all r.m.s. differences are smaller than the observational error of 9 m yr^−1^. We choose *E*=1.25 to avoid overestimating the vertical shearing in slow-flowing areas.

Second, we calibrate the hybrid model by tuning the exponent of the pseudoplastic sliding law *q*, which connects bed-parallel basal shear stress and basal sliding, and the exponent flow law *n* for the SSA. For the SIA, we choose the default value *n*=3. To keep the parameter space manageable we test selected pairs of *q* and *n* (Supplementary Table 3). Here we use the r.m.s. difference along all profiles as our metric. The r.m.s. differences for all experiments are listed in Supplementary Table 3. By this metric, Experiment 7 with (*q*, *n*)=(0.6, 3.25) scores highest. Therefore, all subsequent simulations are performed with (*q*, *n*)=(0.6, 3.25).

## Additional information

**How to cite this article:** Aschwanden, A. *et al.* Complex greenland outlet glacier flow captured. *Nat. Commun.* 7:10524 doi: 10.1038/ncomms10524 (2016).

## Supplementary Material

Supplementary Information Supplementary Figures 1-13, Supplementary Tables 1-4, Supplementary Note 1 and Supplementary References.
