# Supplementary material for: Complex Greenland outlet glacier flow captured
Source: Nat Commun. 2016 Feb 1;7:10524. doi: 10.1038/ncomms10524 (PMC4740423; doi:10.1038/ncomms10524)
Supplement: Supplementary Information — Supplementary Figures 1-13, Supplementary Tables 1-4, Supplementary Note 1 and Supplementary References. [file ncomms10524-s1.pdf]

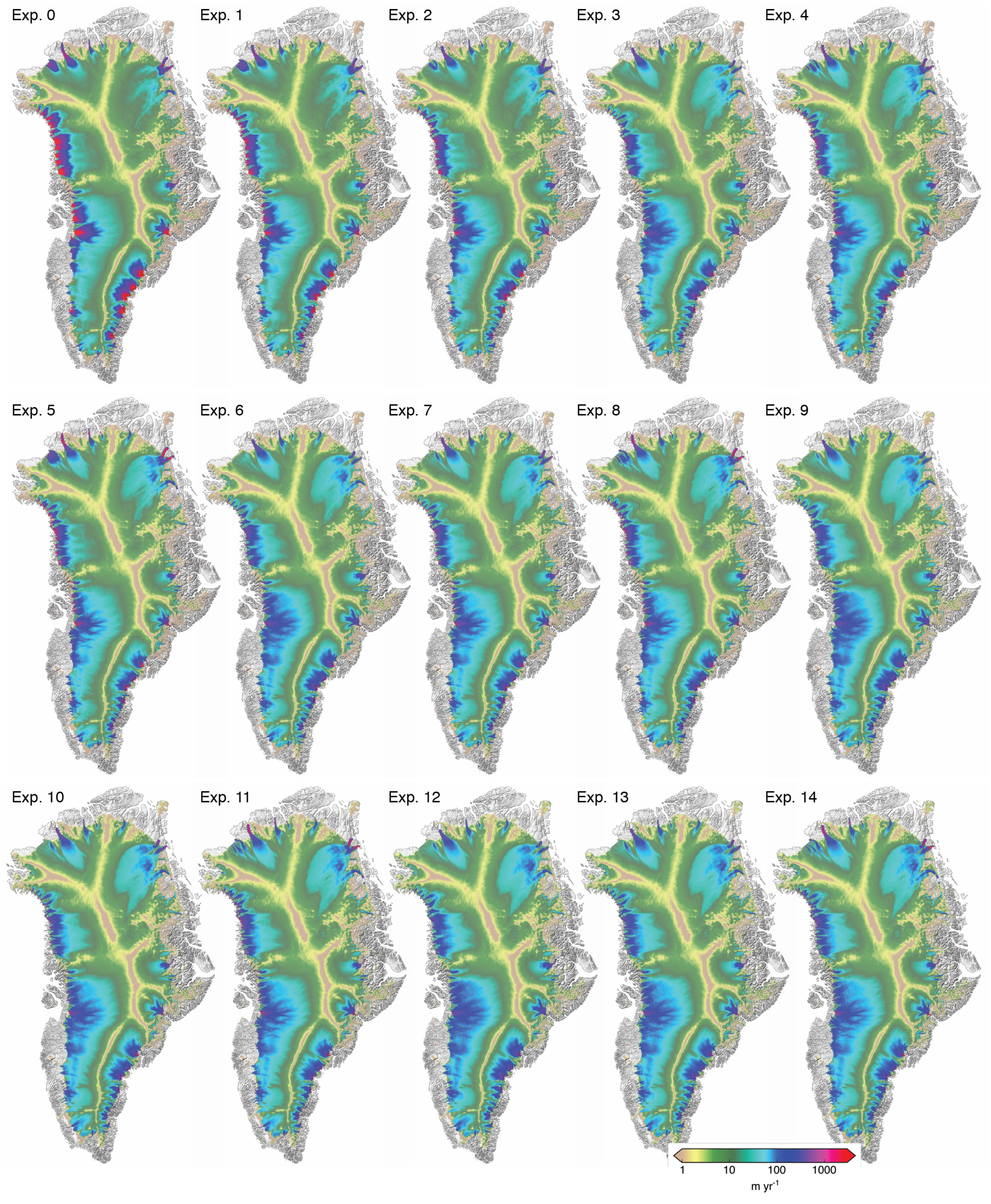

Supplementary Figure 1: Simulated surface speeds of all calibration experiments listed in Supplementary Table 3.

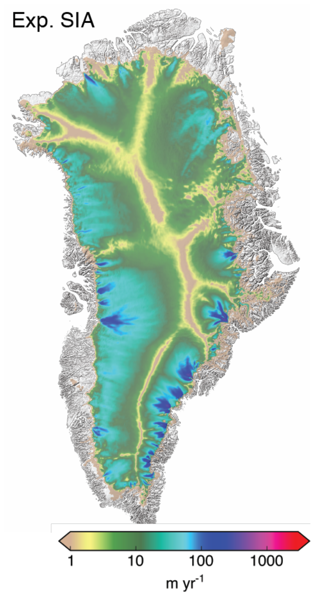

Supplementary Figure 2: Simulated surface speeds using the Shallow Ice Approximation as stress balance.

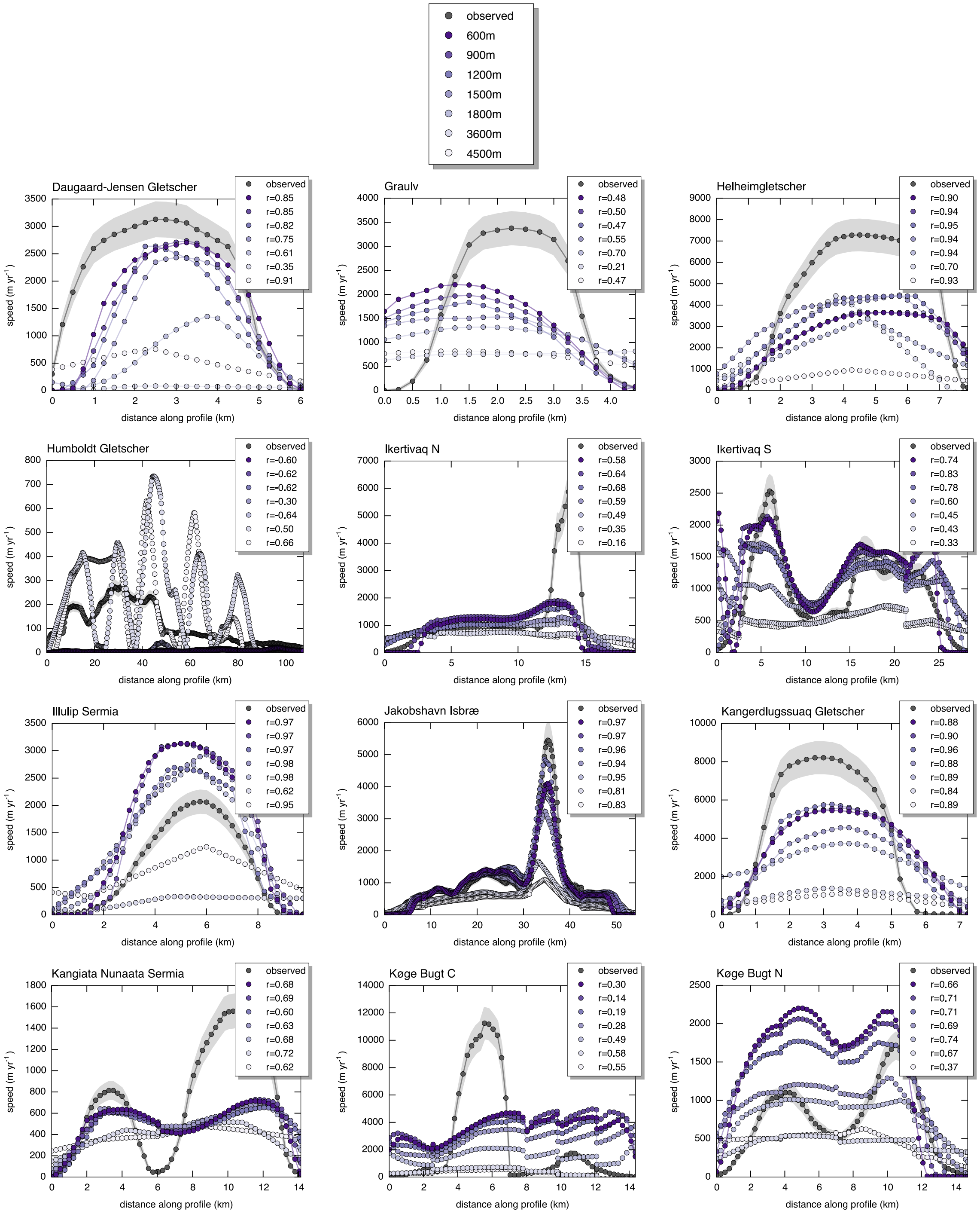

Supplementary Figure 3: Observed and simulated velocity profiles as a function of grid resolution. Continued in Supplementary Figure 4.

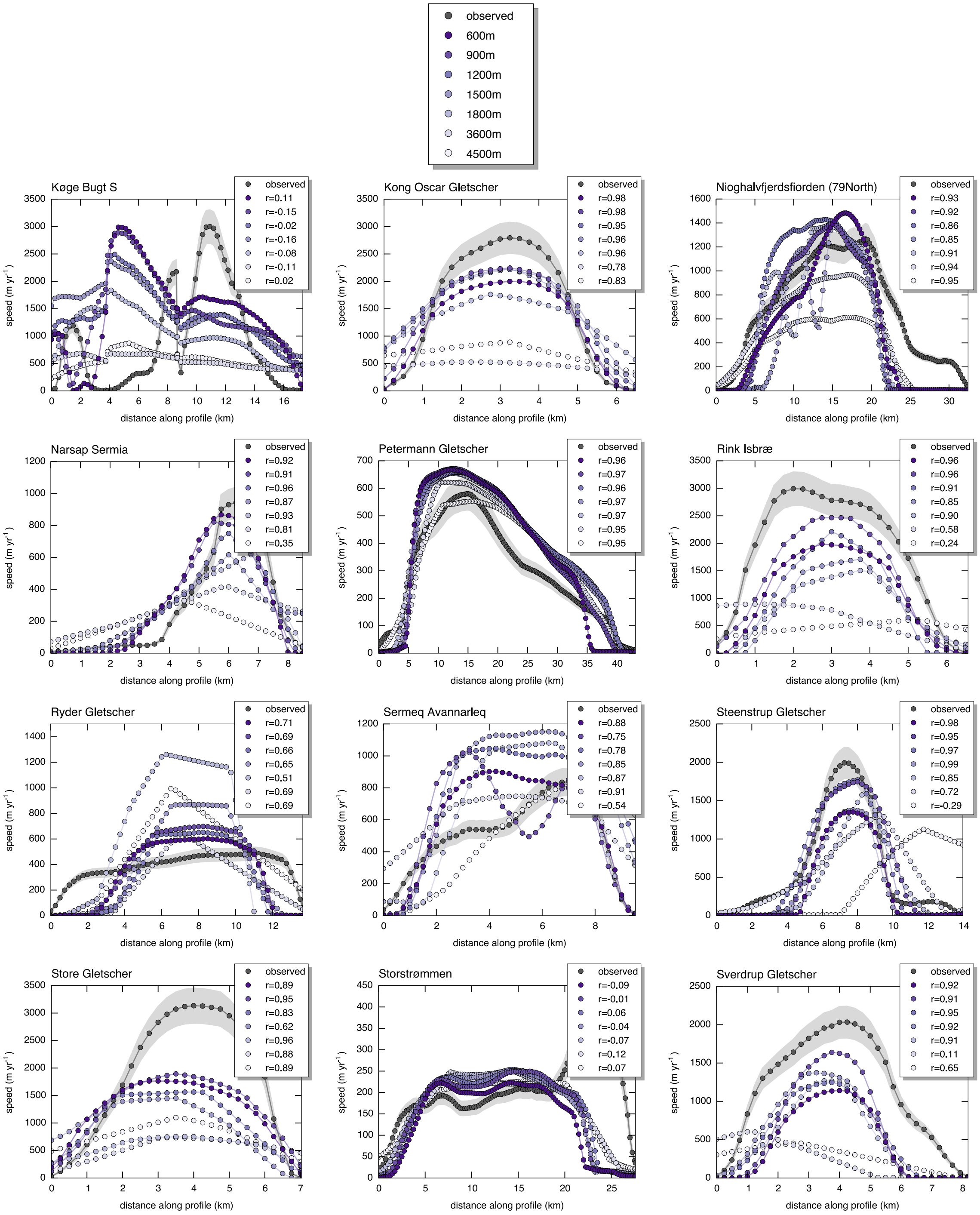

Supplementary Figure 4: Continued from Supplementary Figure 3. Observed and simulated velocity profiles as a function of grid resolution. Continued in Supplementary Figure 5

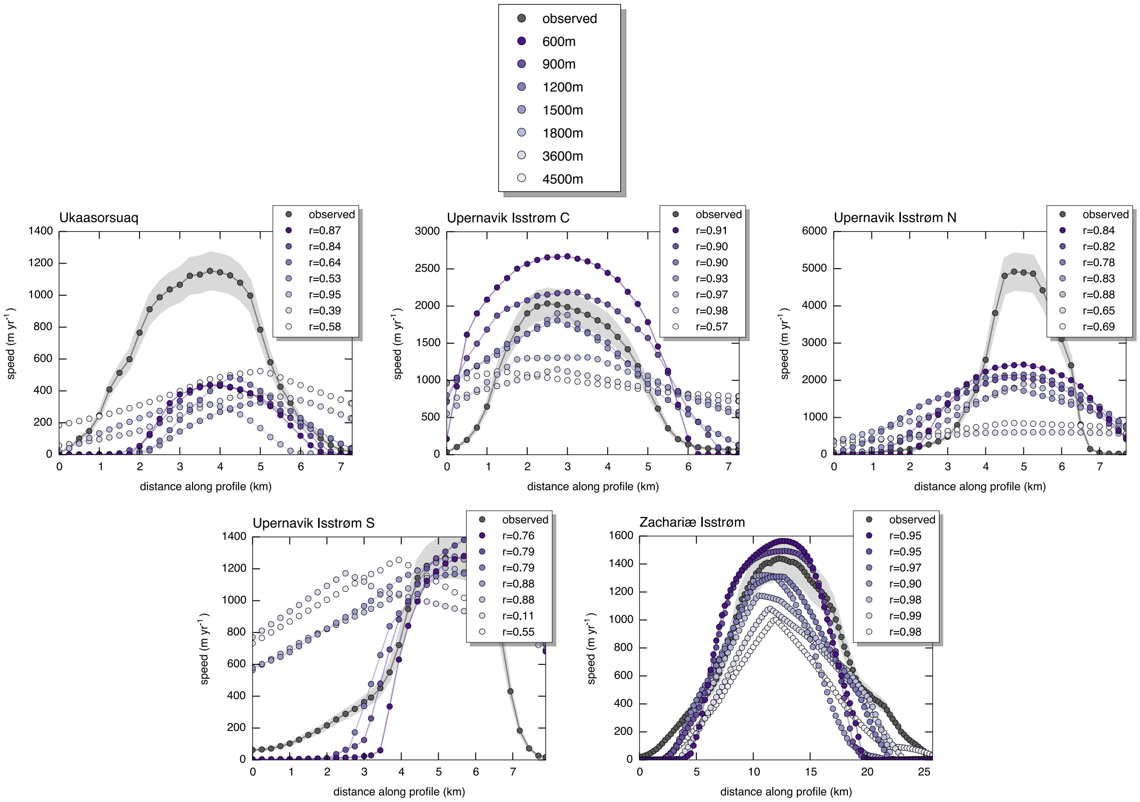

Supplementary Figure 5: Continued from Supplementary Figure 4. Observed and simulated velocity profiles as a function of grid resolution.

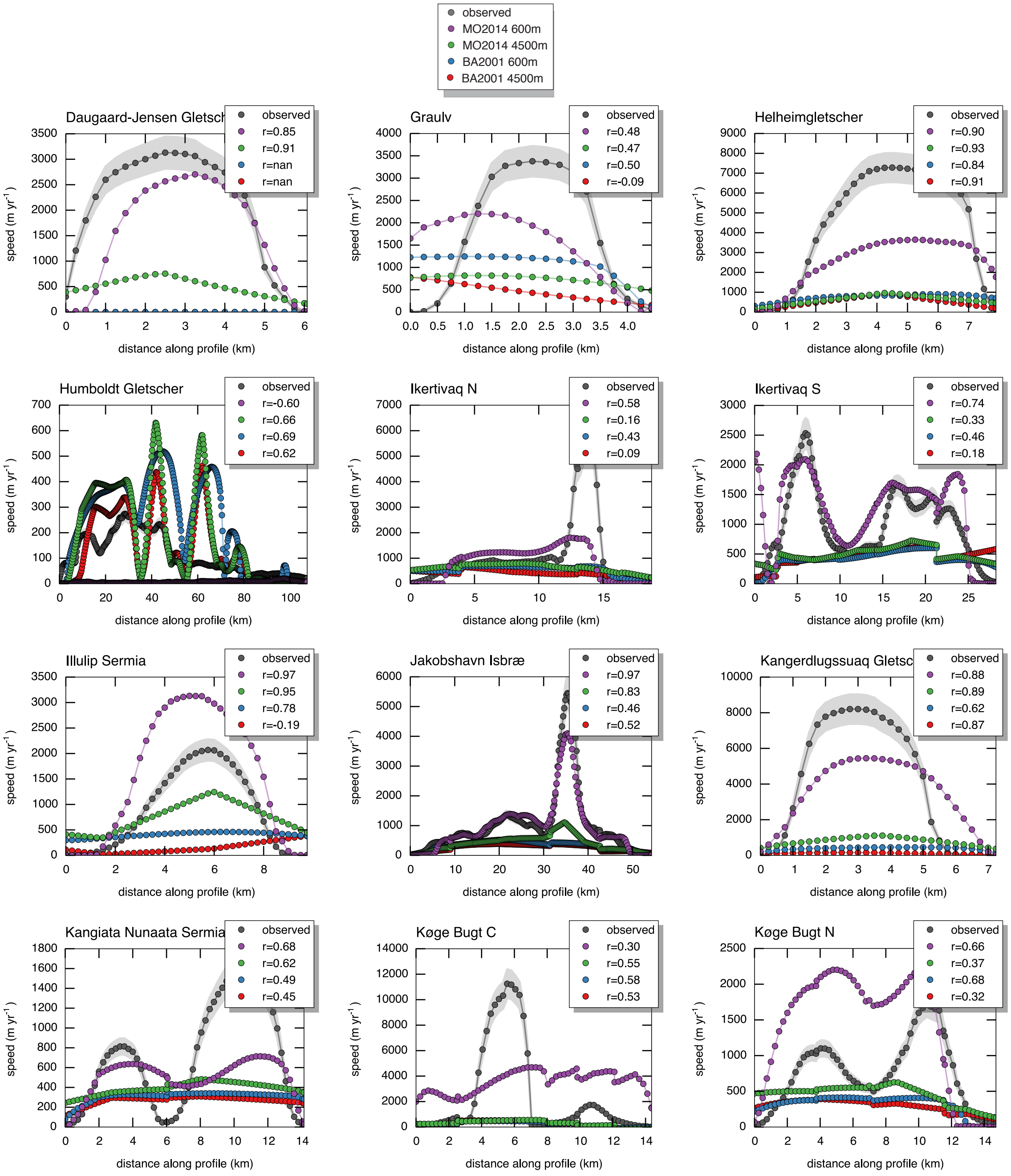

Supplementary Figure 6: Observed and simulated velocity profiles. Experiments with MO2014 bed map[1] at 600 m resolution (MO2014 600 m), MO2014 bed map[1] at 4500 m resolution (MO2014 4500 m), pre-OIB bed map[2] at 600 m model resolution (BA2001 600 m) and 4500 m model resolution (BA2001 4500 m). Continued in Supplementary Figure 7.

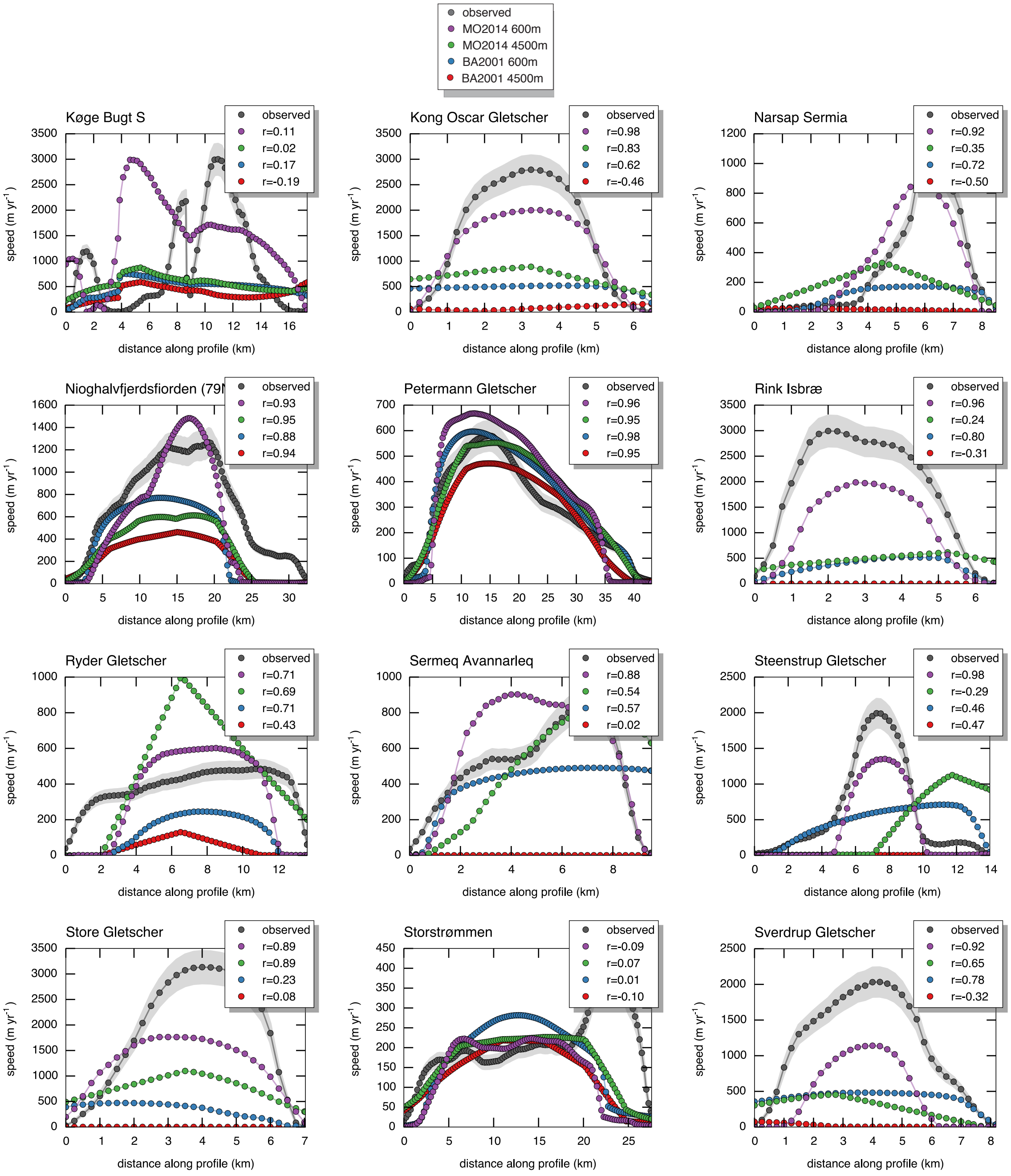

Supplementary Figure 7: Continued from Supplementary Figure 6. Experiments with MO2014 bed map[1] at 600 m resolution (MO2014 600 m), MO2014 bed map[1] at 4500 m resolution (MO2014 4500 m), pre-OIB bed map[2] at 600 m model resolution (BA2001 600 m) and 4500 m model resolution (BA2001 4500 m). Continued in Supplementary Figure 8.

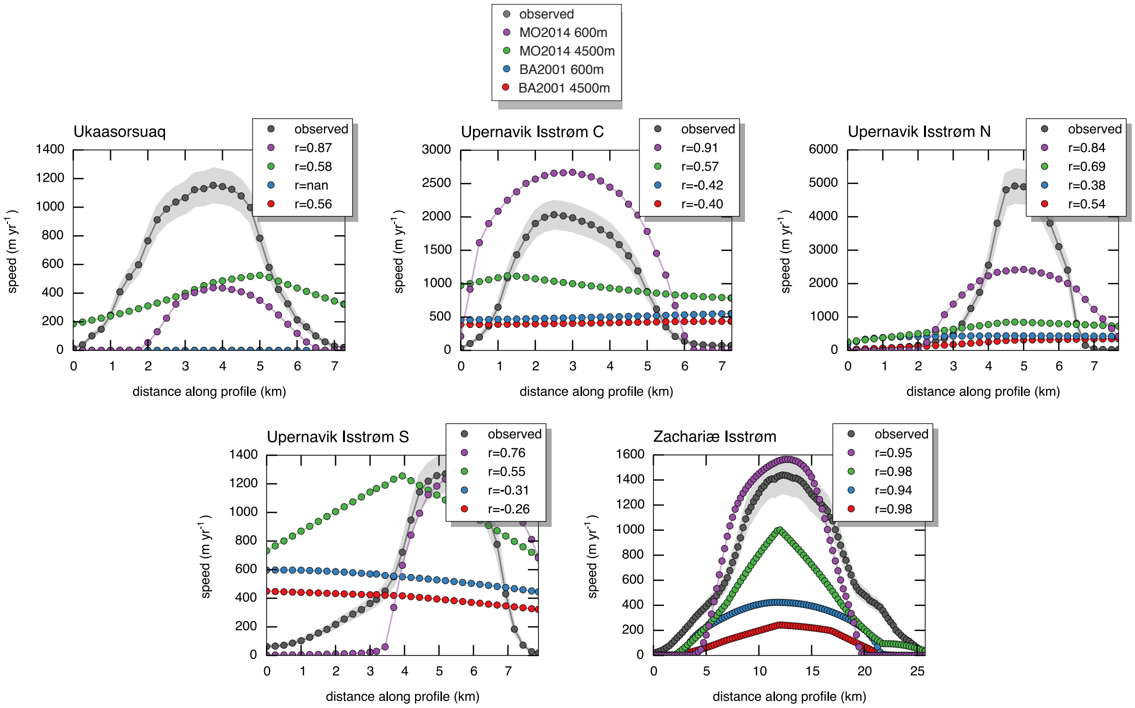

Supplementary Figure 8: Continued from Supplementary Figure 7. Experiments with MO2014 bed map[1] at 600 m resolution (MO2014 600 m), MO2014 bed map[1] at 4500 m resolution (MO2014 4500 m), pre-OIB bed map[2] at 600 m model resolution (BA2001 600 m) and 4500 m model resolution (BA2001 4500 m).

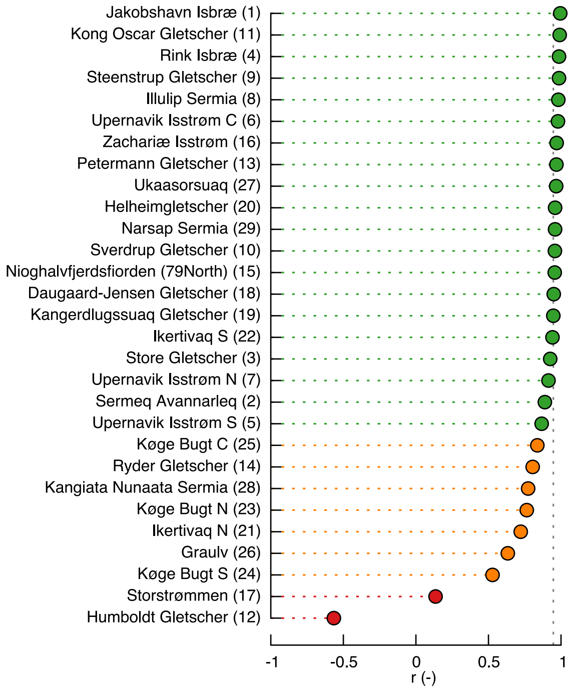

Supplementary Figure 9: Pearson r correlation coefficient between observed and simulated ice flux along cross-flow profiles for the calibrated simulation at 600 m grid resolution. Ice flux is computed as the product of ice thickness and vertically-averaged horizontal velocities. Outlet glacier flow is dominated by longitudinal stretching, we thus assume that vertically-averaged horizontal velocities and horizontal surface velocities are identical.

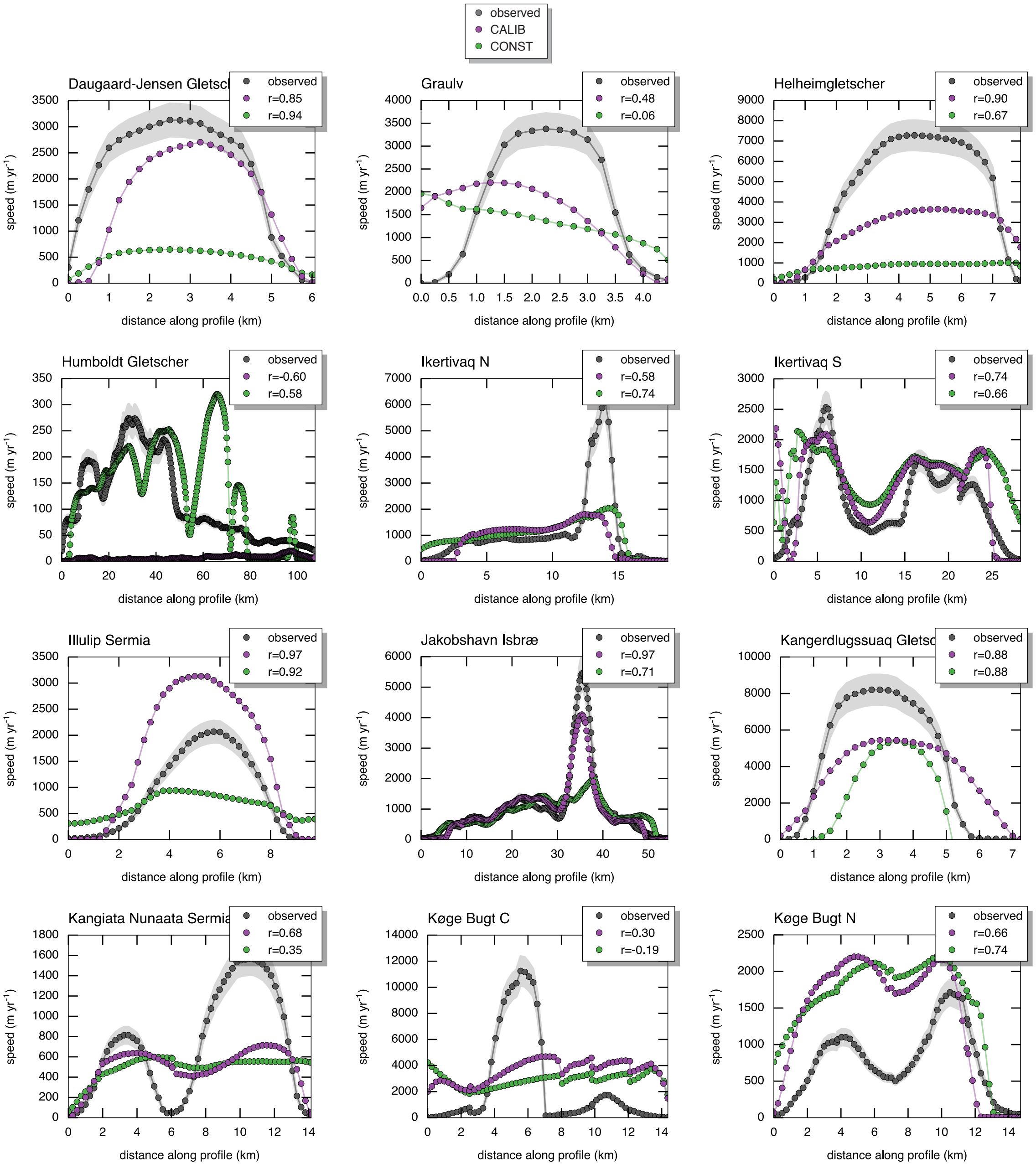

Supplementary Figure 10: Observed and simulated velocity profiles. Calibrated experiment (CALIB) and elevation-independent yield stress (CONST), both with MO2014 bed map[1]. Continued in Supplementary Figure 11.

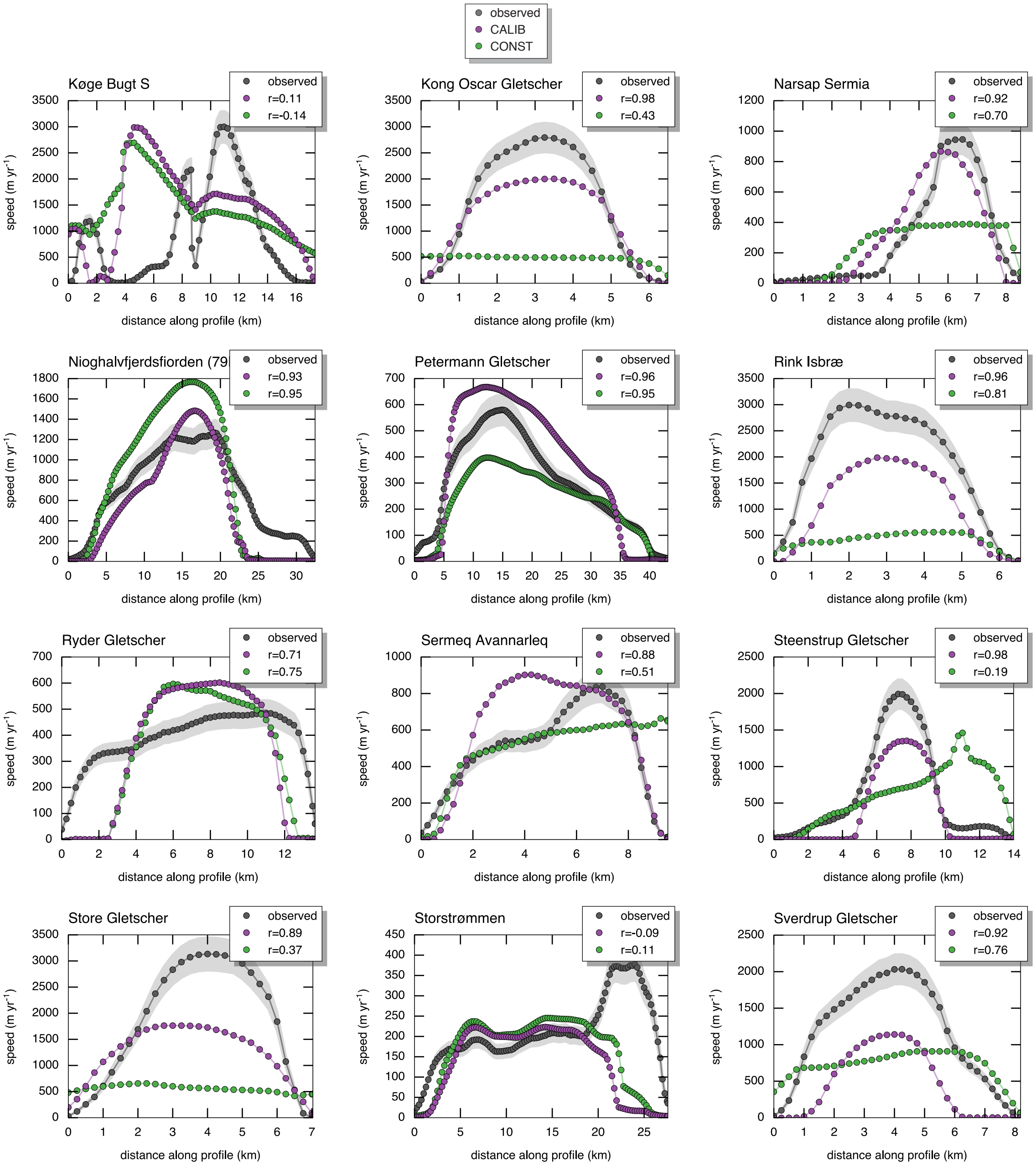

Supplementary Figure 11: Continued from Supplementary Figure 10. Calibrated experiment (CALIB) and elevation-independent yield stress (CONST), both with MO2014 bed map[1]. Continued in Supplementary Figure 12.

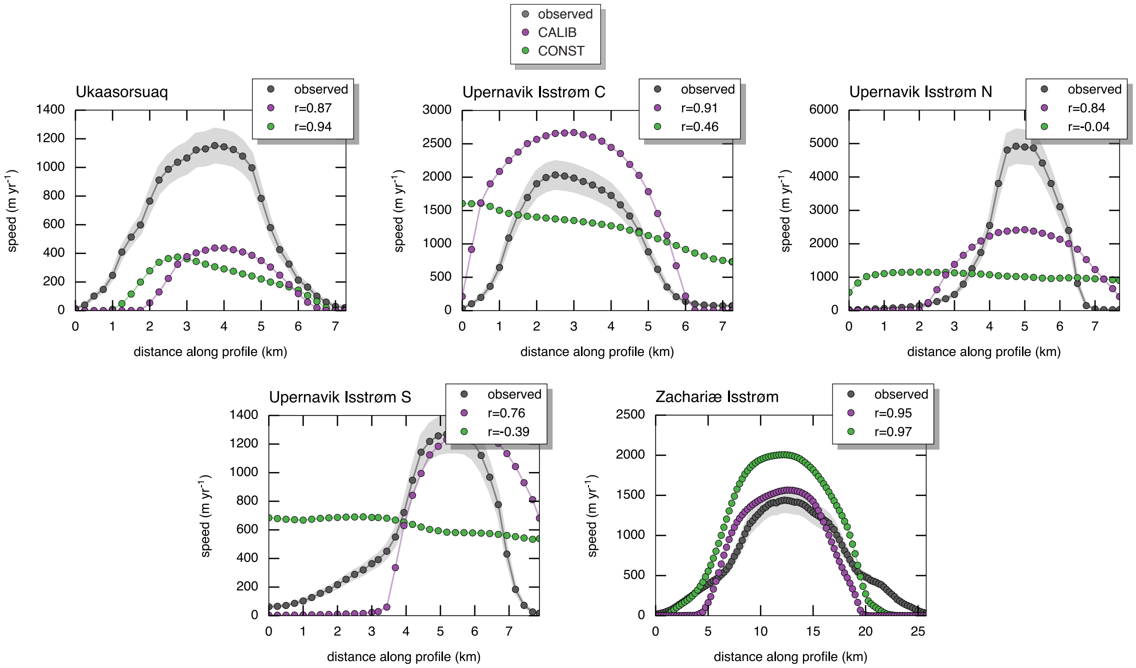

Supplementary Figure 12: Continued from Supplementary Figure 11. Calibrated experiment (CALIB) and elevation-independent yield stress (CONST), both with MO2014 bed map[1].

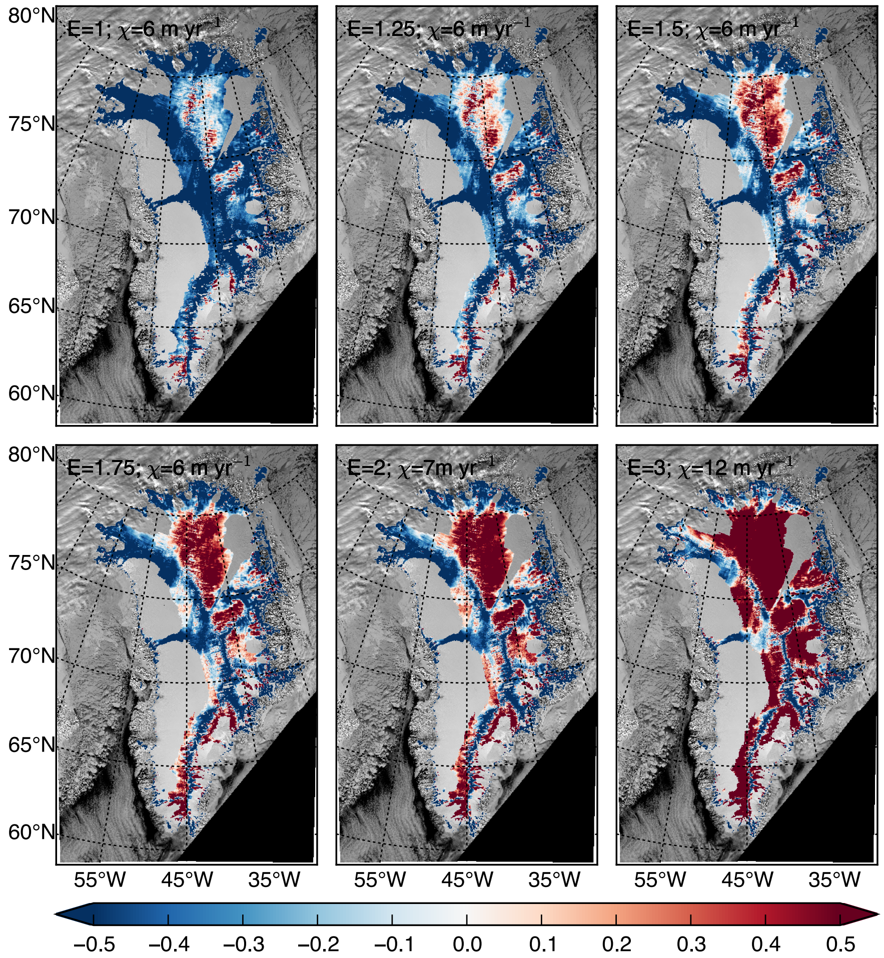

Supplementary Figure 13: Relative difference in surface speeds (model-observation)/observation for different enhancement factors E using the Shallow Ice model. Areas with observed speeds greater than $20\,\mathrm{m\,yr^{-1}}$ are masked.

Supplementary Table 1: List of all simulations. Listed are the horizontal grid resolution in meters (ds); ice thickness/subglacial topography data set (bed) where MO14 (Ref 1) and BA01 (Ref 2); exponent of the SSA flow law (n); exponent of the sliding law (q).

Experiment	ds	bed	n	q
0	1500	MO14	3.00	0.10
1	1500	MO14	3.00	0.25
2	1500	MO14	3.00	0.33
3	1500	MO14	3.00	0.50
4	1500	MO14	3.25	0.50
5	1500	MO14	3.50	0.50
6	1500	MO14	3.00	0.60
7	1500	MO14	3.25	0.60
8	1500	MO14	3.50	0.60
9	1500	MO14	3.00	0.70
10	1500	MO14	3.25	0.70
11	1500	MO14	3.50	0.70
12	1500	MO14	3.00	0.80
13	1500	MO14	3.25	0.80
14	1500	MO14	3.50	0.80
15	600	MO14	3.25	0.60
16	1200	MO14	3.25	0.60
17	1800	MO14	3.25	0.60
18	3600	MO14	3.25	0.60
19	4500	MO14	3.25	0.60
20	600	BA01	3.25	0.60
21	4500	BA01	3.25	0.60

Supplementary Table 2: List of all outlet glaciers for which velocity profiles are calculated, sorted by flux estimated by integrating the product of ice thickness and speed along the profile). Profile length, the glacier type (Ref 3) and the flow type (Ref 4) are also listed. Outlet glacier names are from Ref 5.

Glacier	flux ($\mathrm{Gt\,yr^{-1}}$)	length (km)	glacier type	flow type
Jakobshavn Isbræ	48.5±10.5	54.1	fast-flow marine-term.	isbræ
Helheimgletscher	31.0±6.6	7.9	fast-flow marine-term.	isbræ
Kangerdlugssuaq Gletscher	30.0±6.5	7.2	fast-flow marine-term.	isbræ
Køge Bugt C	15.2±4.1	14.4	fast-flow marine-term.	isbræ
Nioghalvfjerdsfiorden (79North)	12.8±2.8	32.5	ice-shelf-term.	ice-stream
Petermann Gletscher	12.7±2.7	43.1	ice-shelf-term.	isbræ
Zachariæ Isstrøm	12.2±2.7	25.8	ice-shelf-term.	ice-stream
Ikertivaq S	12.0±2.6	28.3	fast-flow marine-term.	isbræ
Rink Isbræ	11.5±2.5	6.6	fast-flow marine-term.	isbræ
Upernavik Isstrøm N	10.9±2.2	7.7	fast-flow marine-term.	isbræ
Store Gletscher	10.6±2.2	7.0	fast-flow marine-term.	isbræ
Kong Oscar Gletscher	10.1±2.2	6.5	fast-flow marine-term.	isbræ
Daugaard-Jensen Gletscher	7.4±1.6	6.1	fast-flow marine-term.	isbræ
Kangiata Nunaata Sermia	6.7±1.4	14.1	fast-flow marine-term.	isbræ
Ikertivaq N	6.4±1.5	18.8	fast-flow marine-term.	isbræ
Illulip Sermia	6.2±1.3	9.8	fast-flow marine-term.	isbræ
Upernavik Isstrom C	6.1±1.3	7.3	fast-flow marine-term.	isbræ
Humboldt Gletscher	5.6±1.4	107.5	fast-flow marine-term.	ice-stream
Sverdrup Gletscher	5.1±1.1	8.2	fast-flow marine-term.	isbræ
Graulv	5.1±1.2	4.4	fast-flow marine-term.	isbræ
Køge Bugt S	4.8±6.5	17.3	fast-flow marine-term.	isbræ
Storstrømmen	4.4±1.1	27.6	low-velocity marine-term.	ice-stream
Steenstrup Gletscher	4.1±1.1	14.0	fast-flow marine-term.	isbræ
Køge Bugt N	4.1±2.7	14.7	fast-flow marine-term.	isbræ
Ryder Gletscher	3.6±0.8	13.6	ice-shelf-term.	isbræ
Upernavik Isstrøm S	3.4±0.8	7.9	fast-flow marine-term.	isbræ
Narsap Sermia	3.3±0.7	8.5	fast-flow marine-term.	isbræ
Ukaasorsuaq	3.2±0.7	7.3	fast-flow marine-term.	isbræ
Sermeq Avannarleq	2.7±0.6	9.5	fast-flow marine-term.	isbræ
Total	299.3±18.2	537.6		

Supplementary Table 3: The rms difference χ between observed and simulated velocity profiles summed over all outlet glaciers of all experiments performed at 1500 m grid resolution. n and q are exponent of the SSA flow law and the sliding law, respectively. χ_{ib} and χ_{is} are the rms differences for all "isbræ"-type and "ice-stream"-type glaciers. $\tilde{r}$ is the median correlation coefficient and $\tilde{r}_{\mathrm{ib}}$ and $\tilde{r}_{\mathrm{is}}$ are the median correlation coefficients of "isbræ"-type and "ice-stream"-type glaciers, respectively.

Exp.	Parameters	$\tilde{r}_{\mathrm{ib}}$ (-)	$\tilde{r}_{\mathrm{is}}$ (-)	$\tilde{r}$ (-)	χ_{ib} (m yr^{-1})	χ_{is} (m yr^{-1})	χ (m yr^{-1})	inc. (%)
7	n=3.25, q=0.60	0.84	0.85	0.85	1188	203	906	
10	n=3.25, q=0.70	0.79	0.86	0.83	1305	181	991	+ 9
3	n=3.00, q=0.50	0.79	0.52	0.74	1302	282	1000	+10
11	n=3.50, q=0.70	0.77	0.90	0.77	1268	468	1005	+11
4	n=3.25, q=0.50	0.80	0.86	0.82	1347	188	1024	+13
14	n=3.50, q=0.80	0.78	0.89	0.78	1318	384	1026	+13
6	n=3.00, q=0.60	0.79	0.51	0.74	1360	292	1043	+15
13	n=3.25, q=0.80	0.79	0.88	0.83	1422	171	1078	+19
9	n=3.00, q=0.70	0.76	0.52	0.75	1439	299	1103	+22
8	n=3.50, q=0.60	0.78	0.93	0.79	1501	525	1183	+31
12	n=3.00, q=0.80	0.74	0.56	0.74	1551	310	1188	+31
2	n=3.00, q=0.33	0.76	0.85	0.77	2215	218	1677	+85
5	n=3.50, q=0.50	0.76	0.91	0.77	2236	665	1742	+92
1	n=3.00, q=0.25	0.73	0.87	0.74	3620	311	2738	+202
0	n=3.00, q=0.10	0.67	0.90	0.69	10722	531	8094	+793

Supplementary Table 4: The rms difference χ between observed and simulated velocity profiles summed over all outlet glaciers of experiments performed at 600 m and 4500 m grid resolution using the MO2014 (Ref 1) and BA2001 (Ref 2) data sets. χ_{ib} and χ_{is} are the rms deiffernces for all "isbræ"-type and "ice-stream"-type glaciers. $\tilde{r}$ is the median correlation coefficient and $\tilde{r}_{\mathrm{ib}}$ and $\tilde{r}_{\mathrm{is}}$ are the median correlation coefficients of "isbræ"-type and "ice-stream"-type glaciers, respectively.

Exp.	Parameters	$\tilde{r}_{\mathrm{ib}}$ (-)	$\tilde{r}_{\mathrm{is}}$ (-)	$\tilde{r}$ (-)	χ_{ib} (m yr^{-1})	inc. (%)	χ_{is} (m yr^{-1})	inc. (%)	χ (m yr^{-1})	inc. (%)
0	bed=MO14, ds=600	0.88	0.93	0.88	1176		168		894	
1	bed=MO14, ds=4500	0.58	0.95	0.62	1673	+42	223	+33	1270	+42
2	bed=BA01, ds=600	0.54	0.88	0.58	1749	+49	267	+59	1331	+49
3	bed=BA01, ds=4500	0.09	0.94	0.25	1837	+56	314	+86	1401	+57

Supplementary Note 1

In some cases, simulations at coarse model and/or data set resolution may produce an apparent improvement. However, this improvement is an artifact of the analysis method. To sample velocities along the cross profiles we use bilinear interpolation (appropriate for a Finite Difference Model). Thus on coarse grids correlation between observed and simulated velocities may be high if the center grid point happens to be flowing faster, as the bilinear interpolation will produce something like a triangle shape flow pattern, similar to channel flow. For example, using the BA2001 data set, correlation coefficients for Helheimgletscher are high, 0.84 and 0.91 at 6000 m and 4500 m, respectively (Supplementary Fig. 7) while the high rms velocity differences clearly show that flow through the Helheimgletscher cross-profile is not well captured by the BA2001 data set.

Supplementary References

1. Morlighem, M., Rignot, E., Mouginot, J., Seroussi, H. & Larour, E. Deeply incised submarine glacial valleys beneath the Greenland ice sheet. *Nature Geoscience* **7**, 18–22 (2014). URL http://www.nature.com/doifinder/10.1038/ngeo2167.

2. Bamber, J. L., Layberry, R. L. & Gogineni, S. P. A new ice thickness and bed data set for the Greenland ice sheet 1. Measurement, data reduction, and errors. *J. Geophys. Res.* **106**, 33773–33780 (2001). URL http://www.agu.org/pubs/crossref/2001/2001JD900054.shtml.

3. Moon, T., Joughin, I., Smith, B. E. & Howat, I. M. 21st-Century Evolution of Greenland Outlet Glacier Velocities. *Science* **693**, 576–578 (2012).

4. Truffer, M. & Echelmeyer, K. A. Of isbræ and ice streams. *Ann. Glaciol.* **36**, 66–72 (2003).

5. Rignot, E. & Mouginot, J. Ice flow in Greenland for the International Polar Year 2008-2009. *Geophysical Research Letters* **39** (2012). URL http://doi.wiley.com/10.1029/2012GL051634.
